# Retraction: LINC00174 facilitates cell proliferation, cell migration and tumor growth of osteosarcoma via regulating the TGF-β/SMAD signaling pathway and upregulating SSH2 expression

**DOI:** 10.3389/fmolb.2023.1208571

**Published:** 2023-05-04

**Authors:** Frontiers Editorial Office

The journal retracts the 17 June 2021 article cited above.

Following publication, concerns were raised regarding the validity of the data in the article. The authors failed to provide the raw data or a satisfactory explanation during the investigation, which was conducted in accordance with Frontiers’ policies. Given the concerns, and the lack of raw data, the editors no longer have confidence in the findings presented in the article.

This retraction was approved by the Chief Editors of Frontiers in Molecular Biosciences and the Chief Executive Editor of Frontiers. The authors have not responded to correspondence regarding this retraction.

